# The frequency-dependent stimulation effects of rTMS on the performance of problem-solving tasks and ongoing oscillations

**DOI:** 10.1192/j.eurpsy.2022.661

**Published:** 2022-09-01

**Authors:** E. Miyauchi, M. Kawasaki

**Affiliations:** University of Tsukuba, Faculty Of Engineering, Tsukuba, Japan

**Keywords:** EEG, TMS, rumination, theta

## Abstract

**Introduction:**

Recent studies suggest that online repetitive transcranial magnetic stimulation (rTMS) can induce local entrainment of ongoing endogenous oscillatory activity that impacts cognitive performance, and the effect may depend on the function of the oscillation. However, little is known about the effects of task-specific frequencies, especially when using an online rTMS paradigm. Our previous electroencephalogram (EEG) study showed that the frontal theta rhythm is associated with the cognitive giving-up processes during problem-solving tasks.

**Objectives:**

In this study, we combined online rTMS and EEG to examine the frequency-dependent stimulation effects of rTMS on the performance of problem-solving tasks and ongoing oscillations. We hypothesized that rTMS at the theta frequency would induce ongoing theta activity and accelerate the giving-up behaviour.

**Methods:**

rTMS was applied during problem-solving tasks with the following conditions: individual theta (4-6Hz)- and alpha (9-13Hz)-TMS, no-TMS, and sham-TMS; the order of conditions was counterbalanced across participants.

**Results:**

Our results showed that theta-frequency rTMS application induced an increase in theta amplitudes and shortened the giving-up response, while a control alpha-frequency rTMS application induced an increase in alpha amplitudes, but did not change giving-up responses.

**Conclusions:**

This study demonstrated the effectiveness of using specific task-relevant stimulation frequency and target location for the modulation of cognitive and behavioral performance. Furthermore, considering the close resemblance between giving-up behaviour and rumination in depression, neuromodulation of cognitive giving-up processes may lead to a new intervention to treat depression by rTMS.

**Disclosure:**

No significant relationships.

